# Social contact patterns in the United Kingdom following the COVID-19 pandemic: The Reconnect cross-sectional survey

**DOI:** 10.1371/journal.pmed.1005038

**Published:** 2026-05-12

**Authors:** Lucy Goodfellow, Billy J. Quilty, Kevin van Zandvoort, W. John Edmunds

**Affiliations:** 1 Centre for the Mathematical Modelling of Infectious Diseases, London School of Hygiene & Tropical Medicine, London, United Kingdom; 2 Charité Centre for Global Health, Charité Universitätsmedizin Berlin, Berlin, Germany; Harvard Medical School, UNITED STATES OF AMERICA

## Abstract

**Background:**

Close-contact and respiratory infectious diseases are spread through social interactions. Measuring these interactions has transformed our ability to understand transmission and control these infections. Social contact patterns were disrupted during the COVID-19 pandemic and have been affected by wider demographic, cultural, and workplace changes since then.

**Methods and findings:**

To estimate post-pandemic social contact patterns in the United Kingdom, we conducted a cross-sectional social contact survey from November 2024 to March 2025 on a nationally representative sample of participants. Interactions were captured by age, gender, and across socioeconomic status (SES) and ethnic groups. We calculated the mean number of daily contacts and contact matrices, stratified by variables of interest, using a negative binomial regression model weighted by age, gender, ethnic group, and weekday/weekend. 13,238 participants were recruited, 3,019 of whom were aged under 18 years old; survey response rates were 36% and 27% for adults and children, respectively. The mean number of daily contacts was 9.1 (95% confidence interval (CI): 8.7, 9.5); this figure was 13.8 (95% CI: 12.8, 14.9) for children, and 7.8 (95% CI: 7.4, 8.2) for adults. Higher numbers of contacts were positively associated with employment, household income, and educational qualifications held. Contact matrices showed high levels of age-assortativity, as well as inter-generational contacts in the home. Contacts were assortative between ethnic groups and SES in all settings; this effect was strongest between ethnic groups in the home, and between SES in the workplace. We constructed socially-stratified next-generation matrices for a novel respiratory pathogen, projecting that the majority White ethnic group would account for the largest share of new infections (76.7% (95% CI: 75.5, 77.9) of cases), but that per-capita infection risk would disproportionately affect minority ethnic groups, with the risk for the Black population being 2.27 (95% CI: 2.06, 2.51) times that of the White population. This study may be limited by the inherent recall biases and reporting fatigue involved with self-reporting contacts.

**Conclusions:**

This study provides crucial data to inform post-pandemic mathematical models of infectious disease transmission, and allows ethnicity and SES to be incorporated in such models.

## Author summary

**Why was this study done?:**

- Data on social contacts are key inputs for mathematical models of infectious disease transmission.
- It is unknown how social contacts have changed since the COVID-19 pandemic, and more generally over the last two decades.
- There is a lack of existing data on social mixing by ethnic and socioeconomic groups, which limits the ability of infectious disease transmission models to investigate inequalities in disease burden.

**What did the researchers do and find?:**

- We conducted a nationally representative cross-sectional survey of over 13,000 people, and collected information on participant demographics and their social contacts.
- On average, people had 9.1 contacts per day, which is a 20% decrease on data from 2006, but a 40% increase from late 2022.
- Mixing was assortative by age group, with the highest daily contacts between children aged 10–14.
- We found that in a disease outbreak in a fully susceptible population, Black people would experience an infection risk 2.3 times that of White people.

**What do these findings mean?:**

- This post-pandemic data on social contacts in the UK can inform and improve models of infectious disease transmission, and expand their capacity beyond age-stratification.
- Social contact patterns may contribute to observed inequalities in infectious disease burden between ethnic groups.
- This study may be affected by people’s ability to accurately recall all of their contacts.

## Abstract 3

In a cross-sectional survey, Lucy Goodfellow and colleagues collect information from over 13,000 people on demographics and their social contacts after the COVID-19 pandemic in the UK.

## Introduction

The control of pathogens which spread through close-contact and respiratory routes, such as influenza, respiratory syncytial virus, and SARS-CoV-2, is imperative for public health. These viruses are transmitted via social interactions, with strong correlations between measured social contacts and pathogen transmission [1–3]. The accuracy of mathematical models of infectious disease transmission has been transformed over the last two decades by the inclusion of data on social contacts. However, many studies which provide such data were performed before the COVID-19 pandemic (e.g., [4–6]), as well as during the pandemic to aid public health responses. The United Kingdom (UK) has experienced large-scale shifts in work and transport patterns since the pandemic, which would be expected to alter social contacts [7,8]; post-pandemic social contact data is needed to investigate this. Furthermore, there is an evidence gap around social contact patterns within and between socioeconomic and ethnic groups, which limits the ability of modelling studies to investigate the impact of interventions on the distribution of diseases across these groups [9,10]. While the importance of this social structuring is well-recognised, its precise impact on epidemic potential is often difficult to quantify due to a lack of sufficiently detailed data capturing contacts between these groups, combined with a lack of stratified epidemiological data. We conducted the Reconnect survey to address these gaps in empirical knowledge, by collecting contemporary data on social interactions across a representative sample of the UK population. We provide updated social contact matrices stratified by age, ethnicity, and socioeconomic status (SES), for the parameterisation of transmission models and study of infectious disease inequalities. Using these matrices, we further quantified infection risk of a novel close-contact pathogen in a completely susceptible population.

## Methods and materials

### Ethics statement

The Reconnect survey received ethical approval from the London School of Hygiene & Tropical Medicine (LSHTM) Research Ethics Committee (LSHTM Ethics Ref: 30212). Formal, informed, written consent was obtained from all participants before survey completion. Formal consent was obtained from the parents/legal guardians of child participants. Participants had the right to withdraw from the study at any time without providing a reason.

### Data management

All data were stored securely in compliance with the Data Protection Act, ensuring participant confidentiality. Anonymised data has been made publicly available to support broader research efforts.

### Study design

We conducted a cross-sectional social contact survey in the UK from 13th December 2024–10th February 2025. Participants were recruited from a UK-based internet panel, using quota sampling to ensure a close representation of the UK population in terms of gender, country, region (as defined by the Office for National Statistics (ONS), within England), household income, and education. We aimed to recruit at least 10,000 adult and 1,000 child participants, deliberately oversampling non-White participants and children in order to reduce data scarcity when conducting stratified analyses. As recruitment rates for children and individuals aged 70+ were lower than expected, we conducted a ‘boost’ round of the survey from 22nd February to 10th March 2025 to increase representation in these age groups.

Participants were invited to the study via email and, upon acceptance, were asked to complete a two-stage prospective diary-based survey using a web page accessible from desktop or mobile devices. They recorded demographic information about themselves and their households in the first stage, and were then instructed to document all contacts they would make on the following day (the ‘survey day’). Participants were encouraged to note down contacts as they occurred throughout the survey day to maximise recall. A contact was defined as someone a participant met in person and whom they spoke with or physically touched. At the end of the survey day, participants completed the second stage of the survey, in which they recorded detailed information for each person they had interacted with. Invitations were distributed evenly across all days of the week to capture representative contact patterns for weekdays and weekends. Participants received a small financial incentive for their participation.

This study is reported as per the Strengthening the Reporting of Observational Studies in Epidemiology (STROBE) guideline (S1 STROBE Checklist). The study protocol is available in S1 Protocol.

### Data collection

For each participant, we collected comprehensive demographic information including age, gender, geographical location (first half of postcode), ethnicity, highest qualification, household income, occupation, housing status, household size, health-related quality of life (using EQ-5D), high-risk status for respiratory diseases, current symptoms, vaccine confidence, and recent public transport use.

For each contact reported, participants recorded the time and setting of the contact (from home, work, school, or other), duration of the contact, whether physical contact occurred, the estimated age, gender, ethnicity, and occupation of the individual contacted, their relationship to the participant, and frequency of typical interaction (days per week). For situations involving numerous contacts that would be difficult to record individually (e.g., large gatherings), participants had the option to record these as aggregate contacts at the end of the questionnaire, with broad estimation of those contact groups’ ages (under 18 years old, 18–64, or over 65), and setting (work, school, and other).

### Sample size

We employed negative binomial regression models to estimate the mean number of daily contacts while accounting for heterogeneity between individuals. Sample size calculations indicated that with 10,000 participants, we would have substantial power to detect a 10% change in the total mean number of daily contacts compared to the POLYMOD survey [4], and the final CoMix surveys [7] (Table A in S1 Text).

### Analysis

#### Data preparation.

Contact data was cleaned and categorised by setting and age groups of participants and their contacts. Participant and contact ages were aggregated into 16 groups: 0−4, 5−9, 10−14, …, 65−69, 70−74, 75+. We defined school holidays as 21st December 2024–5th January 2025, 15th to 23rd February 2025 for participants not living in Wales, and 22nd February–2nd March 2025 for participants living in Wales; all other survey dates were labelled as ‘term time’. Due to an error in data collection, information on whether interactions involved physical contact were not recorded for 4% of contacts. These data were excluded when presenting physical contacts, but included otherwise. Where the same individual was contacted in multiple settings, we assigned their contact setting preferentially to ‘home’ if present, then ‘school’, then ‘work’, then ‘other’. Further details of data cleaning are in S1 Text. We truncated broad age- and setting-specific large group contacts at 300, to reduce the impact of outliers above 300; we also conducted a sensitivity analysis in which the total number of contacts was right-truncated at 100 (Table H in S1 Text).

We determined participants’ SES using the Cascot (Computer Assisted Structured Coding Tool) programme [11]. All participants and contacts with job titles were categorised using the National Statistics Socioeconomic Classification (NS-SEC) as defined by the ONS, which is based on the Standard Occupational Classification 2020 (SOC 2020) and measures employment relations and the conditions of occupations [12]. This classification system aggregates occupations into seven analytic classes, from *Higher managerial, administrative, and professional occupations* to *Routine occupations*, and uses an eighth category for *Never worked and long-term unemployed*, which was not used in this analysis. The additional categories ‘Retired’, ‘Student’, ‘Under 17′, ‘Unemployed’, and ‘Unknown’ were used for participants and contacts without job titles, based on the information provided, where ‘Unemployed’ encompasses all adults who reported not being in employment, but does not differentiate between types of unemployment. Further details of our approach are detailed in S1 Text. We followed the 2021 Census in England and Wales to categorise 19 ethnic groups into five high-level ethnic groups, which we will hereon refer to as ‘Asian’, ‘Black’, ‘Mixed’, ‘White’, and ‘Other’.

#### Survey weighting.

To adjust for potential sampling biases and ensure our sample is representative of the UK population, we applied post-stratification weights with respect to participants’ age group, gender, and ethnicity. These weights were based on the joint age-, gender-, and ethnicity-specific structure in England and Wales, as reported by the 2021 Census [13]. For those who reported their gender as other than male or female, or their ethnicity as ‘Prefer not to say’, no weighting by gender or ethnicity, respectively, was used. Additional weights were used with respect to participants’ survey day, to ensure that weekdays and weekends were represented proportionally. We truncated participants’ weights below and above the 5% and 95% quantiles, respectively, to reduce the impact of outliers with respect to representation.

Analyses of the mean number of contacts stratified by employment status were not weighted by age group due to large differences in the age structure of each strata (Table F in S1 Text). For the same reason, geographically-stratified analyses (including participant’s country) were not weighted by participant ethnicity.

#### Mean contacts.

We assumed that reported contacts follow a negative binomial distribution [4,7,14], and calculated the total and attribute-specific mean number of contacts and associated confidence intervals (CIs) using maximum-likelihood estimation (MLE) with 1,000 bootstrap samples. For each bootstrap sample, participants were sampled with replacement according to the post-stratification weights, and a negative binomial model fitted to the total number of contacts (individually-reported and large group contacts) of the sampled participants. We then reported the mean maximum-likelihood estimate for *μ* (the mean of the negative binomial distribution) and the associated 95% CI, for each of these analyses. We also report dispersion using the inverse of the negative binomial shape parameter, where higher values indicate greater heterogeneity in the number of contacts and values approaching zero approximate a Poisson distribution.

We compared the overall mean number of daily contacts to that found by the POLYMOD survey, which collected data from 1,012 participants in the UK in 2005−2006 [4], and the final CoMix surveys conducted after the acute phase of the COVID-19 pandemic between November and December 2022 when there were no formal restrictions in place on social contacts [7].

#### Contact matrices.

We calculated contact matrices using the mean daily number of contacts between age groups, ethnic groups, and NS-SEC classes, using similar negative binomial MLE for 1,000 bootstrap samples weighted by post-stratification weights. These matrices were stratified by setting: home, work, school, other, and all settings. All contact matrices were weighted with respect to weekday/weekend, while contacts between age groups were further weighted by gender and ethnicity, and contacts between ethnicities were weighted by gender and age group.

In each bootstrap, we fitted negative binomial mean and dispersion parameters to the series of observations of the number of contacts between two groups. The data in each bootstrap was fitted independently, as some contact characteristics were sampled independently in each of the 1,000 bootstraps; this methodology is similar to that of Jarvis and colleagues [7]. We report the mean of the 1,000 fitted parameters.

In the case of age-stratified contact matrices, we distributed participants’ large group contacts to age groups according to the setting-specific contact age distribution found by POLYMOD (adjusted for 2022 age structure) [4]; we chose not to use the individually recorded contact data from this survey as a sampling distribution as it is likely that these large group contacts systematically differ from those individually recorded. In ethnicity- and NS-SEC-stratified contact matrices, we excluded large group contacts. For contacts recorded with ethnicity ‘Prefer not to say’ by a participant of ethnicity *x* (0.93% of contacts), we imputed their ethnicity for the ethnicity-stratified matrix using the distribution of contacts’ ethnicities reported by participants of ethnicity *x*; we repeated this for the NS-SEC class-stratified matrix for contacts whose NS-SEC class was unknown (9.50% of contacts), using distributions based on their corresponding participant’s NS-SEC class. These imputations were sampled independently in each of the 1,000 bootstrap samples used for the contact matrix fitting.

To ensure reciprocity in contacts, i.e., that the number of daily contacts from group *i* to group *j* is equal to the number of daily contacts from group *j* to group *i*, we normalised the age-stratified and ethnicity-stratified matrices with respect to the underlying population structure to produce reciprocal contact matrices, calculating each element by [4,14–16]:


$$\mu_{ij}=\frac{{\hat{\mu }}_{ij}p_{i}+{\hat{\mu }}_{ji}p_{j}}{{\text{2}p}_{i}}$$

where${\hat{\mu }}_{ij}$ is the mean number of daily contacts from group *i* to group *j* in the unadjusted matrix, and $p_{i}$ is the proportion of the population in group *i*, based on Census 2021 data [17]. We present the mean *μ* normalised values.

Assortativity of mixing was calculated using the measure of assortativity *Q*, as described by Keeling and Rohani [18], which was calculated using:


$$Q=\left(\sum_{i=\text{1}}^{n}\left(\frac{\mu_{ii}p_{i}}{\sum_{k=\text{1}}^{n}\mu_{ki}p_{k}}\right)-\text{1}\right)/(n-\text{1})$$

where *n* is the total number of groups in the population. Q=1 describes complete assortativity, Q=0 proportionate mixing, and Q<0 disassortative mixing. We calculated assortativity in overall and setting-specific contact matrices stratified by age group, ethnicity, and NS-SEC class, where NS-SEC class was restricted to interactions between employed individuals, i.e., NS-SEC classes 1–7, to reduce the impact of high assortativity between other groups captured by age groups (under 17s, retired individuals etc.).

#### The effect of social structure on infection risk.

We used socially-stratified next-generation matrices (NGMs) to understand how within- and between-group contact rates and population structure shapes the distribution of infection risk in the early stages of an epidemic of a novel close-contact pathogen (assuming uniform susceptibility, infectiousness, and duration of infectiousness across groups). Analyses were conducted across one-way (age, gender, ethnicity, and NS-SEC) and two-way stratifications (age-ethnicity, age-gender, age–NS-SEC, and ethnicity-NS-SEC).

To calculate NGMs, participants were weighted by weekday/weekend to ensure proportionate representation. For analyses involving multiple demographic stratification variables, only weekday/weekend weighting was applied as census data were not available at that level of granularity due to anonymisation concerns. Values for contacts with unknown ethnicity or NS-SEC class were imputed as above. For gender-specific analyses, we excluded participants and contacts with ‘Other’ gender. We aggregated the Mixed and Other ethnic groups for these analyses, to minimise the effect of small group sizes. Large group contacts were excluded, as ethnicity and NS-SEC data were not collected for these contacts.

The contact rate between groups *i* and $j\left(\mu_{ij}\right)$, representing the average number of contacts an individual in group *i* makes with individuals in group *j*, was adjusted for reciprocity using the same methods as described previously. This reciprocity constraint was applied consistently across all stratification variables (age, ethnicity, NS-SEC, and gender), using the census-derived population denominators for each stratum. The resulting contact matrix *C* is reciprocal $\left(C_{ij}N_{i}=C_{ji}N_{j}\right)$. The NGM was then constructed as $K\propto C^{T}$, where each element $K_{ij}$ represents the expected number of secondary infections in group *i* caused by an infectious individual in group *j*. We used this NGM *K* to derive the dominant right eigenvector (*W*), normalised to sum to 1, which represents the expected distribution of infections across demographic strata during the early exponential growth phase, assuming uniform susceptibility and infectiousness within each stratum. The element $W_{i}$ gives the projected share of infections in stratum *i*. To obtain per-capita infection risk, we divided by population size: $W_{i}/P_{i}$, where $P_{i}$ is based on Census 2021 data.

To compare the per-capita risk of infection across demographic strata, we calculated relative risk as ${RR}_{i}=\left(W_{i}/P_{i}\right)/\left(W_{\text{ref}}/P_{\text{ref}}\right)$ against a reference group. For two-way stratified analyses (e.g., Age × Ethnicity), relative risks were calculated within each age stratum to enable fair comparisons controlling for age effects. Analyses were conducted using 1,000 weighted bootstrap samples.

## Results

### Survey participants

We recruited 13,238 participants, who recorded the characteristics of 50,665 individual and 75,006 large group contacts over a 24-hour period; 11,303 of these participants were recruited in the first study period, and 1,935 in the boost period. 3,019 (22.8%) of our study population were aged under 18 years old, 501 of whom were aged under 5 years old. The survey response rates were 36% for adults, and 27% for children (individuals aged under 18 years old). The median age of the study participants was 38 years old. 54.3% of the study sample was female, 45.5% male, and 0.1% ‘other’. 78.9% of study participants identified their ethnic group as White, 9.5% Black/African/Caribbean/Black British, 8.4% Asian/Asian British, 2.5% Mixed/multiple ethnic groups, 0.6% Other ethnic group, and 0.2% responded with ‘Prefer not to say’. The age breakdown of each ethnicity within the study sample is shown in Fig A in S1 Text, with comparison to data from ONS data on ethnic group-specific age structure. We found a 70% dropoff rate between the demographic survey and the contact survey the following day.

37.9% of the study sample were employed full-time (2.9% self-employed), and 13.7% were employed part-time (1.9% self-employed), while 13% were retired. In comparison, 54.3% of contacts were recorded as employed, 18.2% as not employed (including retired individuals), 20.2% as students, and 7.4% recorded as ‘unknown’. Employed study participants slightly overrepresented NS-SEC classes 1–3, and underrepresented classes 4–7. Further breakdowns of the study sample demography, alongside population proportions from the ONS, are in Table 1.

**Table 1:** Study sample breakdown. Shown by various factors, with comparison to population-level data from the Office for National Statistics (ONS). Some ONS estimates are only calculated using Census 2021 data from England and Wales (ethnicity, highest qualification, employment status, household members, and National Statistics Socioeconomic Classification (NS-SEC) classes), and urban/rural proportions are calculated using data from England only; urban/rural survey proportions exclude participants whose urbanicity was not known.

Variable	Category	% (N)	ONS %
Total		13,238	–
Adult/child	Adult	77.2% (10,219)	79.4%
Adult/child	Child	22.8% (3,019)	20.6%
Age	0–4	3.8% (501)	5.3%
Age	5–9	7.0% (931)	5.8%
Age	10–14	7.4% (977)	6.1%
Age	15–19	6.5% (855)	5.8%
Age	20–24	5.2% (694)	6%
Age	25–29	7.0% (932)	6.5%
Age	30–34	7.6% (1,001)	6.9%
Age	35–39	8.1% (1,070)	6.7%
Age	40–44	7.0% (932)	6.4%
Age	45–49	7.6% (1,005)	6.1%
Age	50–54	7.1% (935)	6.8%
Age	55–59	6.4% (853)	6.8%
Age	60–64	6.1% (804)	6%
Age	65–69	4.7% (620)	5.1%
Age	70–74	4.7% (627)	4.7%
Age	75+	3.8% (501)	9.1%
Gender	Female	54.3% (7,194)	51%
Gender	Male	45.5% (6,026)	49%
Gender	Other	0.1% (17)	–
Ethnicity	White	78.9% (10,439)	81.7%
Ethnicity	Asian	8.4% (1,112)	9.3%
Ethnicity	Black	9.5% (1,255)	4%
Ethnicity	Mixed	2.5% (333)	2.9%
Ethnicity	Prefer not to say	0.2% (21)	–
Ethnicity	Other	0.6% (78)	2.1%
Highest qualification	Apprenticeship	1.9% (258)	4.3%
Highest qualification	Level 1 (1–4 GCSEs, O-levels (any), NVQ level 1, etc.)	8.0% (1,056)	7.9%
Highest qualification	Level 2 (5+ GCSEs, O-levels (passes), NVQ level 2, etc.)	10.9% (1,444)	10.9%
Highest qualification	Level 3 (A-level, BTEC, NVQ level 3, etc.)	12.2% (1,611)	13.8%
Highest qualification	Level 4+ (University degree and above)	39.0% (5,165)	27.5%
Highest qualification	No qualifications	4.9% (652)	14.8%
Highest qualification	Other	0.2% (33)	2.2%
Employment status	Employed full-time (35+ hours per week)	35.0% (4,631)	38.8%
Employment status	Employed part-time	11.8% (1,565)	–
Employment status	Self-employed full-time	2.9% (390)	7.8%
Employment status	Self-employed part-time	1.9% (250)	–
Employment status	Long-term sick or disabled	2.5% (335)	3.4%
Employment status	Looking after home or family	3.0% (395)	3.9%
Employment status	Retired	13.0% (1,722)	17.6%
Employment status	Student	2.5% (336)	4.6%
Employment status	Unemployed (currently looking for work)	2.8% (370)	2.8%
Employment status	Unemployed (not currently looking for work)	1.4% (180)	2.6%
Employment status	Other	0.3% (45)	–
Country	England	85.6% (11,328)	84.5%
Country	Northern Ireland	2.1% (280)	2.8%
Country	Scotland	7.6% (1,007)	8%
Country	Wales	4.7% (623)	4.6%
Household members	1	14.5% (1,922)	28.9%
Household members	2	25.8% (3,409)	20.3%
Household members	3	22.9% (3,030)	21.8%
Household members	4	23.8% (3,147)	9.5%
Household members	5	9.3% (1,234)	3.8%
Household members	6	2.7% (362)	1.6%
Household members	7	0.7% (92)	1.3%
Household members	8+	0.3% (40)	1.3%
NS-SEC class (employed participants)	1 (Higher managerial, administrative and professional occupations)	26.0% (1,731)	15.6%
NS-SEC class (employed participants)	2 (Lower managerial, administrative and professional occupations)	27.9% (1,857)	23.8%
NS-SEC class (employed participants)	3 (Intermediate occupations)	18.2% (1,213)	13.6%
NS-SEC class (employed participants)	4 (Small employers and own account workers)	5.6% (373)	12.6%
NS-SEC class (employed participants)	5 (Lower supervisory and technical occupations)	3.7% (248)	6.4%
NS-SEC class (employed participants)	6 (Semi-routine occupations)	10.5% (697)	13.6%
NS-SEC class (employed participants)	7 (Routine occupations)	8.0% (533)	14.4%
Urban/rural	Rural	12.9% (1,607)	16.9%
Urban/rural	Urban	87.1% (10,895)	83.1%

### Mean contacts

**Fig 1:**
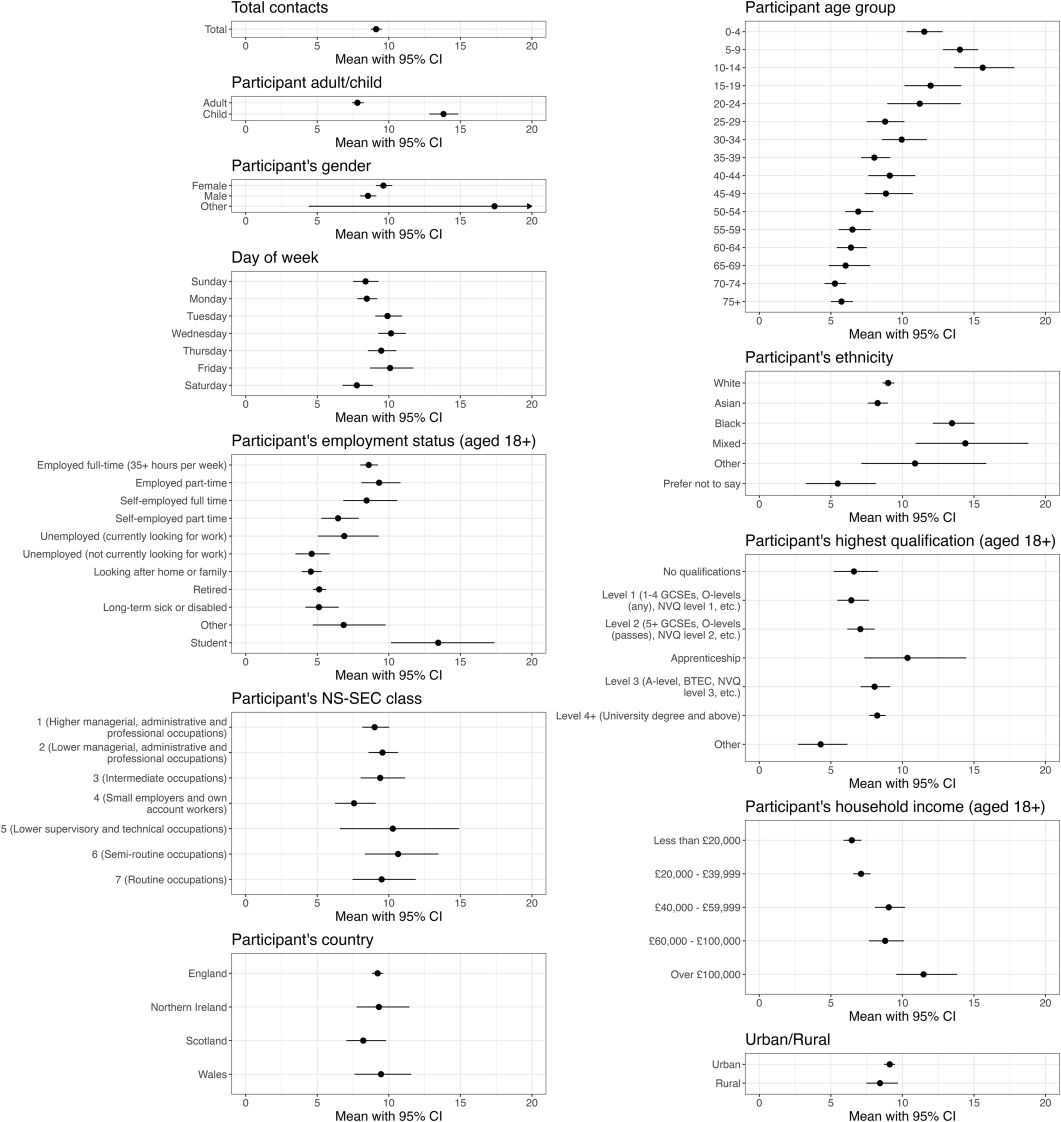
Mean daily number of social contacts and associated 95% confidence intervals. Shown across various stratifications, estimated using a negative binomial regression model weighted by participants’ age group, gender, ethnicity, and weekday/weekend survey day. Models for employment status were not weighted by age, and models for country were not weighted by ethnicity. National Statistics Socioeconomic Classification is denoted by the abbreviation NS-SEC.

The mean number of daily contacts was 9.1 (95% CI: 8.7, 9.5) (Fig 1). This is substantially higher than the mean number of contacts found by Jarvis and colleagues at the end of the COVID-19 pandemic (6.5; 95% CI: 6.0, 7.0) [7], but lower than the mean number of daily contacts found by the POLYMOD survey in 2005−06 (11.7) [4]. We found that the data was overdispersed, therefore justifying the negative binomial assumption over a Poisson distribution, with a mean dispersion parameter estimated via MLE of 1.26 (95% CI: 1.20, 1.33), where high values indicate greater heterogeneity and low values approximate a Poisson distribution. This is greater than the dispersion value of 0.36 found by the POLYMOD survey, and below the value of 1.72 found by Jarvis and colleagues in 2022 [4,7]. We found that 6% of contacts occurred in multiple settings (home/work/school/other). Further comparisons between the three social contact surveys can be found in Table B in S1 Text.

On average, children (hereon defined as participants aged under 18 years old) had 13.8 (95% CI: 12.8, 14.9) daily contacts, compared to 7.8 (95% CI: 7.4, 8.2) for adults. The number of daily contacts increased with age until 10–14 years old, and generally decreased thereafter (Fig 1). The daily number of contacts differed across ethnicities: 9.0 (95% CI: 8.6, 9.4) for participants identifying as White, 13.4 (95% CI: 12.1, 15.0) for those identifying as Black/African/Caribbean/Black British, 8.3 (95% CI: 7.6, 9.0) for Asian/Asian British, 14.4 (95% CI: 10.9, 18.8) for Mixed/multiple ethnic groups, and 10.9 (95% CI: 7.1, 15.9) for Other ethnic groups. While the age structure of each ethnicity differs widely (Fig A in S1 Text), differences in recorded contacts by ethnic group are maintained when standardising by age (Fig B in S1 Text).

Contact levels typically increased with participants’ household income and highest level of qualification, with the exception of those with an apprenticeship (Fig 1). Employed (full- or part-time) or self-employed (full-time) individuals had the greatest number of daily contacts after students; while individuals who were long-term sick or disabled, retired, unemployed and not looking for work, or looking after home or family, the fewest. There was no clear pattern in contact levels when stratified by employed participants’ NS-SEC class. Mean daily contacts typically increased with household size: individuals living alone reported 5.5 (95% CI: 5.1, 6.0) contacts on average, compared to 12.8 (95% CI: 8.2, 18.6) for individuals in households of eight or more people (Table H in S1 Text).

The mean number of daily recorded contacts varied across regions of England, from 8.1 (95% CI: 6.7, 9.8) in the North East to 10.6 (95% CI: 9.4, 12.0) in the East of England. We also found slight variation in the mean number of daily contacts recorded across countries in the UK, from 8.2 (95% CI: 7.2, 9.8) in Scotland to 9.5 (95% CI: 7.7, 11.7) in Wales (Fig F in S1 Text). We did not find a substantial difference in the mean number of daily contacts between participants living in urban and rural areas (Fig 1).

**Fig 2:**
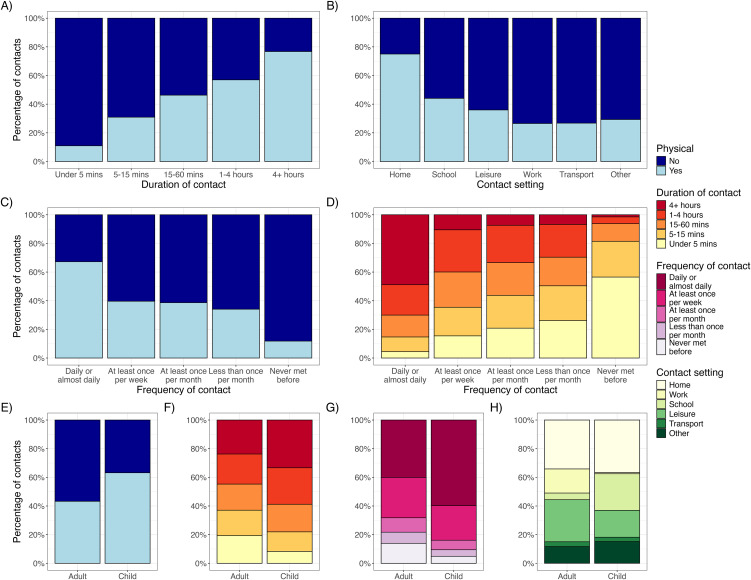
Breakdown of contact types. All proportions weighted by age group, gender, ethnicity, and weekday/weekend. **(A–C)** The proportion of contacts (excluding large group contacts) that involved physical contact, by duration, setting, and frequency of contact. **(D)** The correlation between duration and frequency of contact. **(E–H)** The proportion of contacts in adults and children split by physical contact, duration of contact, frequency of contact, and contact setting. Underlying data for this figure can be found in S2 Table.

Weekdays were associated with 14.8% more contacts for adults than weekends (95% CI: 8.4%, 20.7%), with corresponding figures of 18.9% (95% CI: 10.3%, 26.6%) for children. Contacts of longer duration or increased intensity were more likely to involve physical touch (Fig 2). Seventy-five percentage of contacts occurring in the home involved physical touch, compared to 44% at school and 27% in the workplace. Seventy percentage of contacts made on a daily basis lasted over an hour, whereas 81% of contacts between people who have not met before lasted under 15 minutes.

Children reported substantially more contacts during term time (mean daily contacts: 13.1; 95% CI: 12.4, 13.9) compared to school holidays (9.3; 95% CI: 8.5, 10.3). School holidays were associated with a 29.1% (95% CI: 20.7%, 36.9%) reduction in children’s mean number of contacts. We found similar changes in the mean number of contacts for the elderly but not adults, with relative reductions of 20.2% (95% CI: 6.1%, 31.7%) and 7.8% (95% CI: −8.8%, 23%), respectively (Fig C in S1 Text).

### Contact matrices

**Fig 3:**
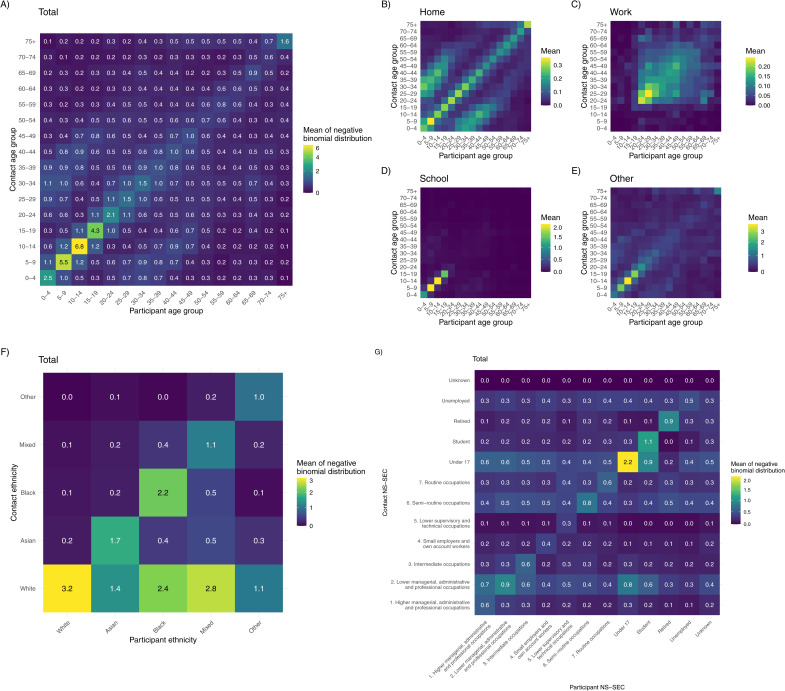
Stratified contact matrices. All contact matrices estimated by the weighted negative binomial regression model. **(A)** Mean number of contacts between individuals of each age group in all settings. **(B–E)** Mean number of contacts between individuals of each age group, stratified by setting. **(F)** Mean number of contacts between individuals of each ethnic group. **(G)** Mean number of contacts between individuals of each National Statistics Socioeconomic Classification (NS-SEC) class or employment categorisation, not adjusted for population-level reciprocity.

**Fig 4:**
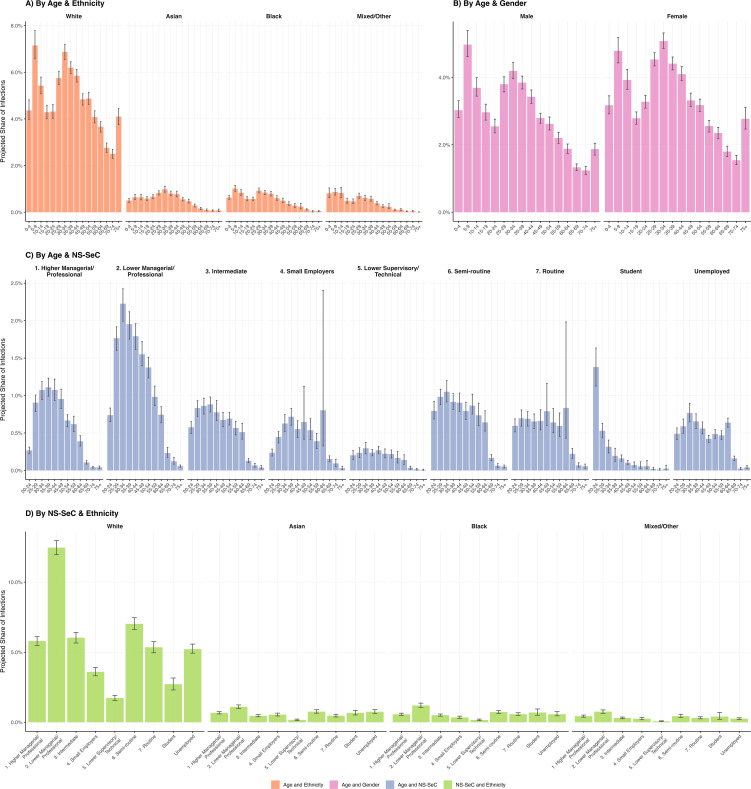
Mean projected share of infections by demographic groups, with 95% confidence intervals. **(A)** Age-ethnicity stratification. **(B)** Age-gender stratification. **(C)** Age-National Statistics Socioeconomic Classification (NS-SEC) stratification, including all NS-SEC categories: working-age categories (1–7), retired, student, under 17, and unemployed. **(D)** Ethnicity-NS-SEC stratification (all ages, working-age categories (1–7), retired, student, under 17, and unemployed).

Contacts were assortative by age group (*Q* = 0.14; 95% CI: 0.13, 0.15), with assortativity strongest in children and in the school setting, and least pronounced in the workplace setting (Figs 3 and I in S1 Text). The highest mean number of contacts between two age groups was between 10–14 year olds (6.71; 95% CI: 5.44, 8.1). Full contact matrix data and confidence intervals, stratified by setting, can be found in S2 Table. There are two parallel diagonals between children and adults, likely reflecting contact with their parents or guardians in the household (Fig 4B). When home contacts are broken down by gender, contacts are both age- and gender-assortative in younger ages, but more disassortative by gender in older ages (Fig J in S1 Text).

The data also displays assortativity between participants’ ethnicity and their contacts’ ethnicity (Q=0.34; 95% CI: 0.32, 0.37), as well as relatively high numbers of White contacts for participants of all ethnicities, driven by over 80% of the UK population identifying as White (Figs 4F and I in S1 Text). Assortativity of contacts by ethnicity is strongest in the home (Figs G and I in S1 Text), but least pronounced in the workplace or school. In the workplace and ‘other’ settings, the majority of all participants’ contacts were White, regardless of the ethnicity of the survey participant. Interactions between NS-SEC classes showed moderate assortativity (*Q* = 0.14; 95% CI: 0.13, 0.16); daily recorded contacts within NS-SEC classes were highest for class 2 (lower managerial, administrative, and professional occupations) and class 6 (semi-routine occupations), respectively, and lowest for class 4 (small employers and own account workers). Assortativity within NS-SEC classes was strongest in the workplace, but less so in other settings (Figs H and I in S1 Text).

### Effect on infection risk

**Fig 5:**
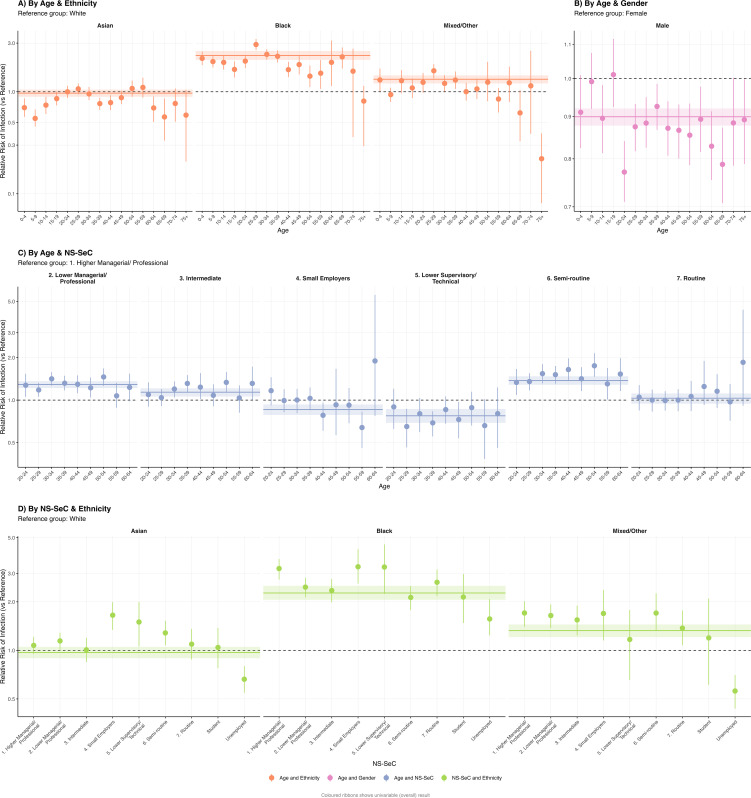
Mean relative risk of infection by demographic groups, with 95% confidence intervals. Relative risks shown on a log scale. Coloured ribbons show univariable mean results with 95% confidence intervals. **(A)** Age-ethnicity stratification, reference group: White. **(B)** Age-gender stratification, reference group: female. **(C)** Age-National Statistics Socioeconomic Classification (NS-SEC) stratification, restricted to the working-age population (ages 20–64, NS-SEC categories 1–7), reference group: 1. Higher Managerial/Professional. **(D)** Ethnicity-NS-SEC stratification, restricted to the working-age population (ages 20–64, NS-SEC categories 1–7), reference group: White.

The largest projected share of infections occurred in the White ethnic group (76.7%, 95% CI: 75.5, 77.9%), and NS-SEC class 2: Lower managerial, administrative, and professional occupations (14.3%, 95% CI: 13.8, 14.8%) (Fig 4). Relative infection risk was highest for the Black (2.27, 95% CI: 2.06, 2.51) and Mixed/Other (1.33, 95% CI: 1.21, 1.45) ethnic groups (compared to the White ethnic group as reference) and the Unemployed (1.51, 95% CI: 1.41, 1.60) NS-SEC groups (compared to NS-SEC class 1: higher managerial and professional occupations as reference) (Fig 5).

In age-stratified analyses, younger and middle-aged groups had the largest projected share of infections across all demographic stratifications. The 5–9, White (7.2%, 95% CI: 6.6, 7.8%) and 30–34, White (6.9%, 95% CI: 6.5, 7.2%) groups were the largest contributors when stratified by age and ethnic group (Fig 4A). We found that the age-specific relative risk (RR) of infection across ethnic groups compared to the White ethnic group varied across age groups; for example, Black 25–29-year-olds were projected to experience a 2.90 (95% CI: 2.58, 3.29) times elevated RR compared to their White counterparts, while the projected RR was 1.42 (95% CI: 1.12, 1.81) for Black 50–54-year-olds (Fig 5A). The same age-dependency in RRs was observed when comparing infection risk between genders (Fig 5B). Minority ethnic groups were projected to experience higher relative infection risks than the White ethnic group within most NS-SEC categories (Fig 5D). Further infection share and RR figures are in Table J in S1 Text and S3 Table.

## Discussion

The accuracy and utility of infectious disease transmission models depend on the availability of accurate data on the population in question. Previously available social contact data do not necessarily reflect the impact of demographic changes and post-pandemic societal structures, and are limited in their ability to accurately describe interactions within and between socioeconomic and ethnic groups. Our study provides up-to-date post-pandemic data on social contact patterns in the UK with a large, representative survey sample across all ages, novel insights into the disparities in social involvement through differences in the mean number of daily contacts across age groups, ethnicities, and socioeconomic groups, and, to the best of our knowledge, the first data on mixing within and between ethnic and socioeconomic groups.

We found that reported contacts have decreased by ~20% since the POLYMOD survey was conducted in 2005–2006, but increased by 40% since late 2022. Mean daily social contacts were greater than those found by POLYMOD in children aged under 5 years old, but lower for all ages above 5; the reduction in contacts was greatest for participants aged 30–50 years old. While contacts were reduced on both weekdays and weekends compared to POLYMOD, we found smaller differences in contact rates between weekdays and weekends for both children and adults than those found by POLYMOD, suggesting a decreased presence of weekday workplace and school contacts. These decreased contact levels suggest that there may be long-term shifts in the epidemiology of a range of close-contact infectious diseases, with corresponding effects on the effectiveness of public health and social measures as well as pharmaceutical interventions such as vaccination programmes. Our overdispersion results indicate that there is substantially greater heterogeneity in contact levels post-pandemic, but less heterogeneity than in 2022.

While the number of recorded social contacts was lower than those found by the POLYMOD survey, age-specific patterns largely followed similar distributions to POLYMOD and other contact surveys, with strong age-assortativity and inter-generational contacts observed. We found that age-assortative contacts in the home are more disassortative by gender in older ages, suggesting that the observed age-assortativity in the home is driven at older ages by heterosexual partnerships. We examined the proportion of contacts which involved physical contact across different settings, durations, and frequencies, which increases risk of transmission; the prevalence of physical contact was very similar to those found by the POLYMOD survey across all stratifications [4]. The proportion of contacts which occurred in multiple settings was also similar to that found by POLYMOD.

A key and novel finding of this study relates to contacts within and between ethnic groups and socioeconomic classes; by comparing assortativity by different attributes across contact settings, this study provides quantitative evidence of assortative mixing between ethnic groups and NS-SEC classes in all settings. While contacts were most assortative between ethnic groups in the home, and between socioeconomic classes in the workplace, these associations were not as strong in other settings. In the workplace or ‘other’ settings, the majority of participants’ contacts were White, regardless of participants’ ethnicity. We found patterns in the mean number of daily contacts across socioeconomic gradients as defined by individuals’ highest level of qualification or household income, and typically higher contact levels for employed individuals than those not in work, but less consistent patterns across NS-SEC classes.

Our analysis suggests that demographic groups projected to have the largest share of infections are not necessarily those with the highest per-capita risk. The largest share of infections was in younger age groups and the White ethnic group (who make up 76.1% (95% CI: 74.8, 77.4) of projected cases), while per-capita infection risk was highest in minority ethnic groups; we found that the Black ethnic group had a relative risk of infection 2.29 (95% CI: 2.08, 2.55) times that of the White ethnic group (reference group). These results suggest that social structure contributes to inequalities in infectious disease burden through differential contact patterns. The inequalities in exposure risk that our findings quantify are consistent with observations during the COVID-19 pandemic, where factors such as over-representation in public-facing occupations and residence in crowded, multi-generational households were drivers of infection risk for ethnic minority communities [19,20]. Our findings suggest that these risk disparities persist within NS-SEC strata, indicating more complex drivers beyond socioeconomic factors. The elevated exposure risk identified through contact patterns could be compounded by other vulnerabilities. Evidence from the pandemic showed that some communities facing higher exposure risk also had greater prevalence of comorbidities that worsened COVID-19 outcomes, and lower vaccination coverage [21–23]. The contact-driven risk we quantify may be one component of morbidity and mortality risk in epidemics and pandemics.

Our data collection methodology had some limitations. Contacts were defined as a contact involving a face-to-face conversation or physical touch. This definition does not necessarily capture all close proximity contacts, such as those in crowded public transport or elevators, or fomite transmission [24]. These transmission events remain difficult to capture through contact diaries. By inferring the age structure of large group contacts according to the setting-specific age distribution of contacts as found by POLYMOD, we implicitly assumed that the age distribution of contacts is similar to that found by the POLYMOD survey, although the magnitude of contacts is assumed to be different. We conducted a sensitivity analysis to investigate the impact of excluding large group contacts on assortativity by age group, and found negligible difference in assortativity (Table I in S1 Text).

Self-reported contacts may have been subject to recall bias and brief interactions with strangers may have been forgotten by participants. We attempted to minimise the effect of recall bias by recruiting individuals one day ahead of the survey date, and prompting participants to write down their interactions through the survey day; 76% of children and 58% of adults reported recording contacts in a list throughout the day. It is also possible that reporting fatigue may have affected participants at higher rates than in the POLYMOD survey, as we asked participants to record more characteristics for each contact. Some of these characteristics may have been estimated by the participant, such as contacts’ age, ethnicity, and occupation, but aggregating information such as ages and ethnicities minimised the impact of estimation. Parents and guardians often filled out the survey on behalf of their child, or helped their child with the survey, potentially leading to lower accuracy for young age groups.

The data analysis was also subject to limitations. Analyses stratified by contacts’ characteristics were limited by the fact that we did not have information on characteristics other than setting and age group for large group contacts, restricting these analyses to individually recorded contacts. Due to an error in data collection, data on physical interaction was missing for 4% of contacts, but we do not believe that this is substantially impactful on data analysis. In our analysis of social mixing within and between socioeconomic groups, we used the NS-SEC classification system. This represented the most pragmatic method of studying contact SES, but is only one definition of SES and does not encompass the wide range of factors which contribute to an individual’s SES. We found disparities in participants’ levels of social interaction by other relevant factors, such as highest level of qualification and household income, but these are difficult to feasibly evaluate for contacts without enrolling all possible contacts in the study. As our socioeconomic classification was based on occupation, it was not possible to analyse contacts across socioeconomic strata within non-working age groups: children and the elderly. Demographic group sizes were estimated using the Census 2021 data for England and Wales, which may not wholly represent the UK population [17]. Census data were not available at sufficient granularity for multi-variable demographic combinations due to anonymisation concerns, preventing full population weighting in multi-variable stratifications. We did not balance NS-SEC-stratified mixing matrices with respect to underlying population sizes, as setting-specific contacts may be seen as a workplace setting by participants but not by their contacts, or vice versa.

There are methodological differences between this survey and previously conducted social contact surveys, which limit direct comparison of results. For example, while this survey and CoMix used online survey methods, the POLYMOD survey used paper diaries [4,7]. In the POLYMOD and Reconnect surveys, participants were first recruited and asked to record their contacts that would occur on the following day (to maximise recall), whereas in CoMix, individuals were asked to recall their contacts from the previous day. Participants in the POLYMOD survey were limited to reporting 30 daily contacts due to the paper diary format, and while they did report data on group contacts, these were not used in the POLYMOD analysis [4]. CoMix did allow for group contacts to be reported, and used a cut-off value of 100 contacts, smaller than our cut-off of 300 contacts per age- and setting-specific group [7]. In both CoMix and this survey, less than 1% of participants reported over 100 contacts.

Our study provides novel data to improve and expand our quantitative understanding of infectious disease transmission, demonstrating disparities in contact levels between various social strata and quantifying mixing patterns by age groups, ethnicity, and socioeconomic classes. Social contact levels are lower than in 2005−2006 but have increased since 2022. This suggests that sociocultural changes in the last two decades have led to lower levels of social interaction, but that as a society, we have returned to levels of interactions that more closely reflect pre-pandemic times than daily life three years ago. This study has also quantified variation in infection risk between ethnic and socioeconomic groups, and demonstrated increased infection risk in minority ethnic groups. These data can inform post-pandemic infectious disease models, which can incorporate ethnicity and SES, to improve understanding of infectious disease transmission and inequalities.

## Supporting information

S1 Text Supplementary materials. **Table A:** Minimum sample sizes required to detect changes in overall contacts compared to previous surveys, given assumptions on magnitude of change and dispersion. **Table B:** Comparison of key features between the POLYMOD, CoMix, and Reconnect social contact surveys [4,7]. **Table C:** Number of contacts reported by participants reporting over 300 contacts in at least one of the broad age- and setting-specific large group contact options, before truncation at 300, and the participants’ age group. **Table D:** NS-SEC class titles, with the number and percentage of the employed survey population in each class, and example job titles for each class. **Table E:** Variables used for weighting in each stratified analysis. **Table F:** Median age in the survey sample, stratified by employment status. **Fig A:** Proportion of the survey sample in each age group, stratified by ethnicity, compared to Census 2021 age- and ethnicity-specific population proportions (dotted lines). Census 2021 data is calculated from England and Wales populations only. **Table G:** The proportion of recorded contacts (excluding large group contacts) taking place in each setting. **Table H:** Total mean number of daily contacts (95% confidence interval), stratified by various variables of interest, as calculated by the weighted negative binomial regression model, and with right-truncation at 100 contacts. Estimates are weighted by participants’ age group, gender, ethnicity, and weekday/weekend survey day, where not already stratified by these variables, except employment status (not weighted by age) and country (not weighted by ethnicity). **Fig B:** Age-standardised mean daily number of social contacts (with 95% confidence interval) across ethnic groups, calculated using the weighted negative binomial regression model. Estimates are weighted by participants’ age group, gender, and weekday/weekend survey day. Age group weighting is applied independently of participant ethnicity, to produce age-standardised results. **Fig C:** Mean daily number of contacts in term time and school holidays, shown for children (aged under 18), adults (aged 18–64), and elderly (aged 65+). All data were weighted by participant age group, gender, ethnicity, and weekday/weekend. **Fig D:** The age distribution of setting-specific contacts, stratified by participant age group, in the POLYMOD survey and this survey (weighted by participant gender, ethnicity, and weekday/weekend, and excluding large group contacts). **Fig E:** Degree distribution of participants, grouped by various participant and contact characteristics. Proportions shown on a log-log scale. **Fig F** Mean daily number of social contacts (with bootstrapped 95% confidence interval) across regions of England and countries in the UK, from the weighted negative binomial regression model. Estimates are weighted by participants’ age group, gender, and weekday/weekend survey day. **Fig G:** Mean number of contacts between individuals of each ethnicity, stratified by setting, as estimated by the weighted negative binomial regression model. **Fig H:** Mean number of contacts between individuals of each NS-SEC class, stratified by setting, as estimated by the weighted negative binomial regression model. These contact matrices are not adjusted for population-level reciprocity. **Fig I:** Mean assortativity of daily contacts between age groups, ethnic groups, and NS-SEC classes (only estimated using contacts between employed individuals, i.e., NS-SEC classes 1–7), using the assortativity measure *Q* as described by Keeling and Rohani [18], where *Q* = 1 describes complete assortativity, *Q* = 0 proportionate mixing, and *Q* = −1 complete disassortative mixing. **Fig J:** Mean number of contacts in the home between individuals of each age group, stratified by gender (restricted to male and female), calculated using the weighted negative binomial regression model (not normalised with respect to age- and gender-specific population structure). **Table I:** Assortativity parameter *Q* for age-specific mixing, by setting of contacts, in the cases of inclusion and exclusion of large group contacts. *Q = 1* describes complete assortativity, *Q = 0* proportionate mixing, and *Q = −1* complete disassortative mixing. **Table J:** One-way stratified analysis results showing projected infection share and risk ratios by demographic group. Risk ratios are calculated relative to reference groups (60–64 age group, White ethnicity, NS-SEC 1, Female gender). Values are means with 95% confidence intervals from 1,000 bootstrap simulations.
(DOCX)

S1 Table Mean number of daily contacts between individuals of each age group, ethnicity, and NS-SEC class, stratified by setting (Total, Home, Work, School, and Other), as estimated by the weighted negative binomial regression model. (XLSX)

S2 Table Multi-variable proportions of daily contacts (by physicality, duration, frequency, setting, and adult/child). (CSV)

S3 Table One- and two-way stratified analysis results showing projected infection share and risk ratios by demographic group. (XLSX)

S1 STROBE Checklist Completed STROBE checklist. An Explanation and Elaboration article discusses each checklist item and gives methodological background and published examples of transparent reporting. The STROBE checklist is best used in conjunction with this article (freely available on the Web sites of PLoS Medicine at http://www.plosmedicine.org/, Annals of Internal Medicine at http://www.annals.org/, and Epidemiology at http://www.epidem.com/). Information on the STROBE Initiative is available at www.strobe-statement.org.
(DOCX)

S1 Protocol Prospective protocol for the study. (PDF)

## Abbreviations

CI: confidence interval
LSHTM: London School of Hygiene & Tropical Medicine
MLE: maximum-likelihood estimation
NGMs: next-generation matrices
NS-SEC: National Statistics Socioeconomic Classification
ONS: Office for National Statistics
RR: relative risk
SES: socioeconomic status
SOC 2020: Standard Occupational Classification 2020
STROBE: Strengthening the Reporting of Observational Studies in Epidemiology.
