# Supplementary material for: Social contact patterns in the United Kingdom following the COVID-19 pandemic: The Reconnect cross-sectional survey
Source: PLoS Med. 2026 May 12;23(5):e1005038. doi: 10.1371/journal.pmed.1005038 (PMC13166901; doi:10.1371/journal.pmed.1005038)
Supplement: S1 STROBE Checklist — An Explanation and Elaboration article discusses each checklist item and gives methodological background and published examples of transparent reporting. The STROBE checklist is best used in conjunction with this article (freely available on the Web sites of PLoS Medicine at http://www.plosmedicine.org/, Annals of Internal Medicine at http://www.annals.org/, and Epidemiology at http://www.epidem.com/). Information on the STROBE Initiative is available at www.strobe-statement.org. (DOCX) [file pmed.1005038.s005.docx]

STROBE Statement—checklist of items that should be included in reports of observational studies

|  | Item No. | Recommendation | Page  No. | Relevant text from manuscript |
| --- | --- | --- | --- | --- |
| **Title and abstract** | 1 | (*a*) Indicate the study’s design with a commonly used term in the title or the abstract | Abstract, Methods and Findings section | we conducted a cross-sectional social contact survey from November 2024 to March 2025 on a nationally representative sample of participants |
|  |  | (*b*) Provide in the abstract an informative and balanced summary of what was done and what was found | Abstract, Methods and Findings section | To estimate post-pandemic social contact patterns in the United Kingdom, we conducted a cross-sectional social contact survey from November 2024 to March 2025 on a nationally representative sample of participants. Interactions were captured by age, gender, and across socioeconomic status (SES) and ethnic groups. We calculated the mean number of daily contacts and contact matrices, stratified by variables of interest, using a negative binomial regression model weighted by age, gender, ethnic group, and weekday/weekend. 13,238 participants were recruited, 3,019 of whom were aged under 18 years old. The mean number of daily contacts was 9.1 (95% confidence interval (CI): 8.7, 9.5); this figure was 13.8 (95% CI: 12.8, 14.9) for children, and 7.8 (95% CI: 7.4, 8.2) for adults. Higher numbers of contacts were positively associated with employment, household income, and educational qualifications held. Contact matrices showed high levels of age-assortativity, as well as intergenerational contacts in the home. Contacts were assortative between ethnic groups and SES in all settings; this effect was strongest between ethnic groups in the home, and between SES in the workplace. We constructed socially stratified next-generation matrices for a novel respiratory pathogen, projecting that the majority White ethnic group would account for the largest share of new infections (76.7% (95% CI: 75.5, 77.9) of cases), but that per-capita infection risk would disproportionately affect minority ethnic groups, with the risk for the Black population being 2.27 (95% CI: 2.06, 2.51) times that of the White population. |
| Introduction | | | |  |
| Background/rationale | 2 | Explain the scientific background and rationale for the investigation being reported | Introduction, paragraph 1 | many studies which provide such data were performed before the COVID-19 pandemic (e.g. [[4–6]](https://www.zotero.org/google-docs/?XUvqpR)), as well as during the pandemic to aid public health responses. The United Kingdom (UK)K has experienced large-scale shifts in work and transport patterns since the pandemic, which would be expected to alter social contacts [[7,8)]](https://www.zotero.org/google-docs/?RV3Czu); post-pandemic social contact data is needed to investigate this. Furthermore, there is an evidence gap around social contact patterns within and between socioeconomic and ethnic groups, which limits the ability of modelling studies to investigate the impact of interventions on the distribution of diseases across these groups [[9,10]](https://www.zotero.org/google-docs/?S2YOWa). |
| Objectives | 3 | State specific objectives, including any prespecified hypotheses | Introduction, paragraph 1 | We conducted the Reconnect survey to address these gaps in empirical knowledge, by collecting contemporary data on social interactions across a representative sample of the UK population. We provide updated social contact matrices stratified by age, ethnicity, and socioeconomic status (SES), for the parameterisation of transmission models and study of infectious disease inequalities. Using these matrices, we further quantified infection risk of a novel close-contact pathogen in a completely susceptible population. |
| Methods | | | |  |
| Study design | 4 | Present key elements of study design early in the paper | Methods and Materials, Study design, paragraph 1 | We conducted a cross-sectional social contact survey in the UK from 13th December 2024 to 10th February 2025. |
| Setting | 5 | Describe the setting, locations, and relevant dates, including periods of recruitment, exposure, follow-up, and data collection | Methods and Materials, Study design, paragraph 1 | We conducted a cross-sectional social contact survey in the UK from 13th December 2024 to 10th February 2025. Participants were recruited from a UK-based internet panel, using quota sampling to ensure a close representation of the UK population in terms of gender, country, region (as defined by the Office for National Statistics (ONS), within England), household income, and education. We aimed to recruit at least 10,000 adult and 1,000 child participants, deliberately oversampling non-White participants and children in order to reduce data scarcity when conducting stratified analyses. As recruitment rates for children and individuals aged 70+ were lower than expected, we conducted a second round of the survey from 22nd February to 10th March 2025 to increase representation in these age groups. |
| Participants | 6 | (*a*) *Cohort study*—Give the eligibility criteria, and the sources and methods of selection of participants. Describe methods of follow-up  *Case-control study*—Give the eligibility criteria, and the sources and methods of case ascertainment and control selection. Give the rationale for the choice of cases and controls  *Cross-sectional study*—Give the eligibility criteria, and the sources and methods of selection of participants | NA |  |
|  |  | (*b*) *Cohort study*—For matched studies, give matching criteria and number of exposed and unexposed  *Case-control study*—For matched studies, give matching criteria and the number of controls per case | NA |  |
| Variables | 7 | Clearly define all outcomes, exposures, predictors, potential confounders, and effect modifiers. Give diagnostic criteria, if applicable | Methods and Materials, Study design, paragraph 2  Analysis, Data Preparation, paragraph 1 and 2 | A contact was defined as someone a participant met in person and whom they spoke with or physically touched.  Participant and contact ages were aggregated into 16 groups: 0-4, 5-9, 10-14, …, 65-69, 70-74, 75+.  We determined participants’ SES using the Cascot (Computer Assisted Structured Coding Tool) programme [[11]](https://www.zotero.org/google-docs/?VpAY68). All participants and contacts with job titles were categorised using the National Statistics Socioeconomic Classification (NS-SeC) as defined by the ONS, which is based on the Standard Occupational Classification 2020 (SOC 2020) and measures employment relations and the conditions of occupations [[12]](https://www.zotero.org/google-docs/?zYeoaH). This classification system aggregates occupations into seven analytic classes, from *Higher managerial, administrative, and professional occupations* to *Routine occupations*, and uses an eighth category for *Never worked and long-term unemployed*, which was not used in this analysis. The additional categories ‘Retired’, ‘Student’, ‘Under 17’, ‘Unemployed’, and ‘Unknown’ were used for participants and contacts without job titles, based on the information provided, where ‘Unemployed’ encompasses all adults who reported not being in employment, but does not differentiate between types of unemployment. Further details of our approach are detailed in Supplementary Section 5. We followed the 2021 Census in England and Wales to categorise 19 ethnic groups into five high-level ethnic groups, which we will hereon refer to as ‘Asian’, ‘Black’, ‘Mixed’, ‘White’, and ‘Other’. |
| Data sources/ measurement | 8* | For each variable of interest, give sources of data and details of methods of assessment (measurement). Describe comparability of assessment methods if there is more than one group | NA |  |
| Bias | 9 | Describe any efforts to address potential sources of bias | Analysis, survey weighting, paragraph 1 | To adjust for potential sampling biases and ensure our sample is representative of the UK population, we applied post-stratification weights with respect to participants’ age group, gender, and ethnicity. These weights were based on the joint age-, gender-, and ethnicity-specific structure in England and Wales, as reported by the 2021 Census [[13]](https://www.zotero.org/google-docs/?u8VnSr). |
| Study size | 10 | Explain how the study size was arrived at | Analysis, Sample size | We employed negative binomial regression models to estimate the mean number of daily contacts while accounting for heterogeneity between individuals. Sample size calculations indicated that with 10,000 participants, we would have substantial power to detect a 10% change in the total mean number of daily contacts compared to the POLYMOD survey [[4]](https://www.zotero.org/google-docs/?JdyyWo), and the final CoMix surveys [[7]](https://www.zotero.org/google-docs/?UZHWjf) (Supplementary Section 1). |

| Quantitative variables | 11 | Explain how quantitative variables were handled in the analyses. If applicable, describe which groupings were chosen and why | Analysis, data preparation | Contact data was cleaned and categorised by setting and age groups of participants and their contacts. Participant and contact ages were aggregated into 16 groups: 0-4, 5-9, 10-14, …, 65-69, 70-74, 75+. We defined school holidays as 21st December 2024 to 5th January 2025, 15th to 23rd February 2025 for participants not living in Wales, and 22nd February - 2nd March 2025 for participants living in Wales; all other survey dates were labelled as ‘term time’. Due to an error in data collection, information on whether interactions involved physical contact were not recorded for 4% of contacts. These data were excluded when presenting physical contacts, but included otherwise. Where the same individual was contacted in multiple settings, we assigned their contact setting preferentially to ‘home’ if present, then ‘school’, then ‘work’, then ‘other’. Further details of data cleaning are in Supplementary Section 4. We truncated broad age- and setting-specific large group contacts at 300, to reduce the impact of outliers above 300; we also conducted a sensitivity analysis in which the total number of contacts was right-truncated at 100 (Supplementary Section 8b).  We determined participants’ SES using the Cascot (Computer Assisted Structured Coding Tool) programme [[11]](https://www.zotero.org/google-docs/?VpAY68). All participants and contacts with job titles were categorised using the National Statistics Socioeconomic Classification (NS-SeC) as defined by the ONS, which is based on the Standard Occupational Classification 2020 (SOC 2020) and measures employment relations and the conditions of occupations [[12]](https://www.zotero.org/google-docs/?zYeoaH). This classification system aggregates occupations into seven analytic classes, from *Higher managerial, administrative, and professional occupations* to *Routine occupations*, and uses an eighth category for *Never worked and long-term unemployed*, which was not used in this analysis. The additional categories ‘Retired’, ‘Student’, ‘Under 17’, ‘Unemployed’, and ‘Unknown’ were used for participants and contacts without job titles, based on the information provided, where ‘Unemployed’ encompasses all adults who reported not being in employment, but does not differentiate between types of unemployment. Further details of our approach are detailed in Supplementary Section 5. We followed the 2021 Census in England and Wales to categorise 19 ethnic groups into five high-level ethnic groups, which we will hereon refer to as ‘Asian’, ‘Black’, ‘Mixed’, ‘White’, and ‘Other’. |
| --- | --- | --- | --- | --- |
| Statistical methods | 12 | (*a*) Describe all statistical methods, including those used to control for confounding | Analysis, Mean contacts | We assumed that reported contacts follow a negative binomial distribution [[4,7,14]](https://www.zotero.org/google-docs/?bfc840), and calculated the total and attribute-specific mean number of contacts and associated confidence intervals (CIs) using maximum-likelihood estimation (MLE) with 1000 bootstrap samples.  For each bootstrap sample, participants were sampled with replacement according to the post-stratification weights, and a negative binomial model fitted to the total number of contacts (individually-reported and large group contacts) of the sampled participants. We then reported the mean maximum likelihood estimate for μ (the mean of the negative binomial distribution) and the associated 95% CI, for each of these analyses. |
|  |  | (*b*) Describe any methods used to examine subgroups and interactions | NA |  |
|  |  | (*c*) Explain how missing data were addressed | Analysis, Contact matrices | For contacts recorded with ethnicity ‘Prefer not to say’ by a participant of ethnicity *x* (0.93% of contacts), we imputed their ethnicity for the ethnicity-stratified matrix using the distribution of contacts’ ethnicities reported by participants of ethnicity *x*; we repeated this for the NS-SEC class-stratified matrix for contacts whose NS-SEC class was unknown (9.50% of contacts), using distributions based on their corresponding participant’s NS-SEC class. These imputations were sampled independently in each of the 1000 bootstrap samples used for the contact matrix fitting. |
|  |  | (*d*) *Cohort study*—If applicable, explain how loss to follow-up was addressed  *Case-control study*—If applicable, explain how matching of cases and controls was addressed  *Cross-sectional study*—If applicable, describe analytical methods taking account of sampling strategy | Analysis, Survey weighting | To adjust for potential sampling biases and ensure our sample is representative of the UK population, we applied post-stratification weights with respect to participants’ age group, gender, and ethnicity. |
|  |  | (*e*) Describe any sensitivity analyses | NA |  |
| Results | | | | |
| Participants | 13* | (a) Report numbers of individuals at each stage of study—eg numbers potentially eligible, examined for eligibility, confirmed eligible, included in the study, completing follow-up, and analysed | Results, Survey participants, paragraph 1 | We recruited 13,238 participants, who recorded the characteristics of 50,665 individual and 75,006 large group contacts over a 24-hour period; 11,303 of these participants were recruited in the first study period, and 1,935 in the boost period. |
|  |  | (b) Give reasons for non-participation at each stage | NA |  |
|  |  | (c) Consider use of a flow diagram | NA |  |
| Descriptive data | 14* | (a) Give characteristics of study participants (eg demographic, clinical, social) and information on exposures and potential confounders | Results, Survey participants, paragraph 1-2 | 3,019 (22.8%) of our study population were aged under 18 years old, 501 of whom were aged under 5 years old. The median age of the study participants was 38 years old. 54.3% of the study sample was female, 45.5% male, and 0.1% ‘other’. 78.9% of study participants identified their ethnic group as White, 9.5% Black/African/Caribbean/Black British, 8.4% Asian/Asian British, 2.5% Mixed/multiple ethnic groups, 0.6% Other ethnic group, and 0.2% responded with ‘Prefer not to say’. The age breakdown of each ethnicity within the study sample is shown in Figure S2, with comparison to data from ONS data on ethnic group-specific age structure. We found a 70% dropoff rate between the demographic survey and the contact survey the following day.  37.9% of the study sample were employed full-time (2.9% self-employed), and 13.7% were employed part-time (1.9% self-employed), while 13% were retired. In comparison, 54.3% of contacts were recorded as employed, 18.2% as not employed (including retired individuals), 20.2% as students, and 7.4% recorded as ‘unknown’. Employed study participants slightly overrepresented NS-SEC classes 1-3, and underrepresented classes 4-7. Further breakdowns of the study sample demography, alongside population proportions from the ONS, are in Table 1. |
|  |  | (b) Indicate number of participants with missing data for each variable of interest | Table 1 | - |
|  |  | (c) *Cohort study*—Summarise follow-up time (eg, average and total amount) | NA |  |
| Outcome data | 15* | *Cohort study*—Report numbers of outcome events or summary measures over time | NA |  |
|  |  | *Case-control study—*Report numbers in each exposure category, or summary measures of exposure |  |  |
|  |  | *Cross-sectional study—*Report numbers of outcome events or summary measures | Results, Mean contacts, paragraph 1 | The mean number of daily contacts was 9.1 (95% CI: 8.7, - 9.5) (Figure 1). |
| Main results | 16 | (*a*) Give unadjusted estimates and, if applicable, confounder-adjusted estimates and their precision (eg, 95% confidence interval). Make clear which confounders were adjusted for and why they were included | Results, Mean contacts, paragraph 1 | The mean number of daily contacts was 9.1 (95% CI: 8.7, - 9.5) (Figure 1). |
|  |  | (*b*) Report category boundaries when continuous variables were categorized | NA |  |
|  |  | (*c*) If relevant, consider translating estimates of relative risk into absolute risk for a meaningful time period | NA |  |

| Other analyses | 17 | Report other analyses done—eg analyses of subgroups and interactions, and sensitivity analyses | Results, Mean contacts, paragraph 2 | On average, children (hereon defined as participants aged under 18 years old) had 13.8 (95% CI: 12.8, 14.9) daily contacts, compared to 7.8 (95% CI: 7.4, 8.2) for adults. The number of daily contacts increased with age until 10-14 years old, and generally decreased thereafter (Fig. 1). The daily number of contacts differed across ethnicities: 9.0 (95% CI: 8.6, 9.4) for participants identifying as White, 13.4 (95% CI: 12.1, 15.0) for those identifying as Black/African/Caribbean/Black British, 8.3 (95% CI: 7.6, 9.0) for Asian/Asian British, 14.4 (95% CI: 10.9, 18.8) for Mixed/multiple ethnic groups, and 10.9 (95% CI: 7.1,  15.9) for Other ethnic groups. |
| --- | --- | --- | --- | --- |
| Discussion | | | | |
| Key results | 18 | Summarise key results with reference to study objectives | Discussion, paragraph 2 | We found that reported contacts have decreased by approximately 20% since the POLYMOD survey was conducted in 2005-06, but increased by 40% since late 2022. |
| Limitations | 19 | Discuss limitations of the study, taking into account sources of potential bias or imprecision. Discuss both direction and magnitude of any potential bias | Discussion, paragraphs 6-8 | Our data collection methodology had some limitations. Contacts were defined as a contact involving a face-to-face conversation or physical touch. This definition does not necessarily capture all close proximity contacts, such as those in crowded public transport or elevators, or fomite transmission [[24]](https://www.zotero.org/google-docs/?JcyMeS). These transmission events remain difficult to capture through contact diaries. By inferring the age structure of large group contacts according to the setting-specific age distribution of contacts as found by POLYMOD, we implicitly assumed that the age distribution of contacts is similar to that found by the POLYMOD survey, although the magnitude of contacts is assumed to be different. We conducted a sensitivity analysis to investigate the impact of excluding large group contacts on assortativity by age group, and found negligible difference in assortativity (Supplementary Section 8f).  Self-reported contacts may have been subject to recall bias and brief interactions with strangers may have been forgotten by participants. We attempted to minimise the effect of recall bias by recruiting individuals one day ahead of the survey date, and prompting participants to write down their interactions through the survey day; 76% of children and 58% of adults reported recording contacts in a list throughout the day. It is also possible that reporting fatigue may have affected participants at higher rates than in the POLYMOD survey, as we asked participants to record more characteristics for each contact. Some of these characteristics may have been estimated by the participant, such as contacts’ age, ethnicity, and occupation, but aggregating information such as ages and ethnicities minimised the impact of estimation. Parents and guardians often filled out the survey on behalf of their child, or helped their child with the survey, potentially leading to lower accuracy for young age groups.  The data analysis was also subject to limitations. Analyses stratified by contacts’ characteristics were limited by the fact that we did not have information on characteristics other than setting and age group for large group contacts, restricting these analyses to individually-recorded contacts. Due to an error in data collection, data on physical interaction was missing for 4% of contacts, but we do not believe that this is substantially impactful on data analysis. In our analysis of social mixing within and between socioeconomic groups, we used the NS-SeC classification system. This represented the most pragmatic method of studying contact SES, but is only one definition of SES and does not encompass the wide range of factors which contribute to an individual’s SES. We found disparities in participants’ levels of social interaction by other relevant factors, such as highest level of qualification and household income, but these are difficult to feasibly evaluate for contacts without enrolling all possible contacts in the study. As our socioeconomic classification was based on occupation, it was not possible to analyse contacts across socioeconomic strata within non-working age groups: children and the elderly. Demographic group sizes were estimated using the Census 2021 data for England and Wales, which may not wholly represent the UK population [[17]](https://www.zotero.org/google-docs/?ttbzpS). Census data were not available at sufficient granularity for multi-variable demographic combinations due to anonymisation concerns, preventing full population weighting in multi-variable stratifications. We did not balance NS-SEC-stratified mixing matrices with respect to underlying population sizes, as setting-specific contacts may be seen as a workplace setting by participants but not by their contacts, or vice versa. |
| Interpretation | 20 | Give a cautious overall interpretation of results considering objectives, limitations, multiplicity of analyses, results from similar studies, and other relevant evidence | Discussion, final paragraph | Our study provides novel data to improve and expand our quantitative understanding of infectious disease transmission, demonstrating disparities in contact levels between various social strata and quantifying mixing patterns by age groups, ethnicity, and socioeconomic classes. Social contact levels are lower than in 2005-06 but have increased since 2022. This suggests that sociocultural changes in the last two decades have led to lower levels of social interaction, but that as a society, we have returned to levels of interactions that more closely reflect pre-pandemic times than daily life three years ago. |
| Generalisability | 21 | Discuss the generalisability (external validity) of the study results | Discussion, paragraph 1 | Our study provides up-to-date post-pandemic data on social contact patterns in the UK with a large, representative survey sample across all ages, novel insights into the disparities in social involvement through differences in the mean number of daily contacts across age groups, ethnicities, and socioeconomic groups, and, to the best of our knowledge, the first data on mixing within and between ethnic and socioeconomic groups. |
| Other information | |  | | |
| Funding | 22 | Give the source of funding and the role of the funders for the present study and, if applicable, for the original study on which the present article is based | Acknowledgements, Funding | This work was supported by the National Institute for Health and Care Research (NIHR) Health Protection Research Unit in Modelling and Health Economics, a partnership between the UK Health Security Agency, Imperial College London and LSHTM (grant code NIHR200908, www.nihr.ac.uk). BJQ and WJE were supported by the NIHR Health Protection Research Unit in Modelling and Health Economics (grant code NIHR200908, www.nihr.ac.uk). The funders had no role in study design, data collection and analysis, decision to publish, or preparation of the manuscript. The views expressed are those of the author(s) and not necessarily those of the NIHR, UK Health Security Agency or the Department of Health and Social Care. |

*Give information separately for cases and controls in case-control studies and, if applicable, for exposed and unexposed groups in cohort and cross-sectional studies.

**Note:** An Explanation and Elaboration article discusses each checklist item and gives methodological background and published examples of transparent reporting. The STROBE checklist is best used in conjunction with this article (freely available on the Web sites of PLoS Medicine at http://www.plosmedicine.org/, Annals of Internal Medicine at http://www.annals.org/, and Epidemiology at http://www.epidem.com/). Information on the STROBE Initiative is available at www.strobe-statement.org.
