# Supplementary material for: Social contact patterns in the United Kingdom following the COVID-19 pandemic: The Reconnect cross-sectional survey
Source: PLoS Med. 2026 May 12;23(5):e1005038. doi: 10.1371/journal.pmed.1005038 (PMC13166901; doi:10.1371/journal.pmed.1005038)
Supplement: S1 Protocol — (PDF) [file pmed.1005038.s006.pdf]

UK Social Contact Survey 2024

Billy J Quilty, W John Edmunds

Background and rationale

Social contacts play a critical role in the transmission dynamics of infectious diseases. Understanding who people are interacting with, how frequently they are doing so, and under what circumstances these interactions occur is essential for predicting the spread of diseases such as COVID-19 and developing targeted interventions to mitigate their impact.

The COVID-19 pandemic significantly altered patterns of social interaction in the UK and elsewhere as evidenced by the CoMix survey conducted during the pandemic. Some of these changes are likely to have persisted after the acute phase of the pandemic due to cultural shifts such as remote working. To better understand current social contact patterns in the UK and their potential implications for infectious disease transmission, we will conduct a comprehensive social contact survey in 2024. As in previous surveys, we will collect detailed demographic information on participants and how they make contacts (quantity, duration, closeness, frequency, etc.). We will also investigate key unknowns not assessed previously in the UK, such as how contacts vary seasonally, the assortativity of contacts by socioeconomic status and ethnicity, and contact with animals, factors which may have substantial implications for transmission and health outcomes.

This research will contribute valuable insights into social contact patterns in the UK and their relationship to infectious disease transmission. By collecting up-to-date data on social contacts and their associated characteristics, this study will support ongoing efforts to protect population health and minimise the burden of infectious diseases.

Aims and Objectives

This study aims to collect detailed data on social contacts in the UK, including key demographic information, to:

- Describe current patterns of social interaction and assess how they have changed compared to previous surveys
- Investigate the extent to which social contacts vary according to factors such as age, gender, socioeconomic status, and ethnicity
- Examine how social contact patterns change seasonally (e.g., winter vs. summer, school terms vs. holidays)
- Identify any notable trends or patterns that could inform the development of future public health interventions
- Create stratified contact matrices to parameterise mathematical models.

Study Design

We will conduct several cross-sectional surveys in the United Kingdom in summer and in winter, covering holiday/non-holiday periods in each (term time/school holidays and pre-Christmas/ Christmas). We will survey both adults and children about their mixing behaviour to fully capture differences in different age groups.

Enrollment Procedure

We aim to survey at least 10,000 individuals in total, split evenly between the summer and winter surveys. We will use a market research company to ensure a sample representative of the national population in terms of age, gender, geographical location, socioeconomic status, and ethnicity via quota sampling. Children enrolled in the survey will be encouraged to complete the survey themselves, and the questions will be asked in such a way as to be understandable to all.

Participants will be invited to the study via email, which will explain the purpose of the study and how they will take part. If they accept, participants will be first asked to record demographic information about themselves (“participant information”). Following this, they will then be instructed to record their contacts made on the following day (the “survey day”) in a prospective diary-based approach using a mobile-accessible app or webpage. On the survey day, participants will be encouraged to make a note of contacts (e.g. their initials) as they happen, or in regular time intervals (e.g. at lunchtime, dinnertime, before bed), to maximise recall. At the end of the survey day, individuals will be asked to then record the detailed demographic information and contact event information for the contacts made on the day (“contact information”).

Participants will be invited until the specified quotas have been filled. Effort should be made to ensure equal spread across the different days of the week to capture work/school week and weekend effects. Participants will be offered a small financial incentive to take part (e.g. Amazon vouchers or similar).

Measurements

Participants will be asked to record the following information about themselves:

- Age
- Gender
- Geographical location (first part of postcode)
- Ethnicity
- Household income
- Occupation
- Highest level of education
- Housing status (homeowner, private renter, etc.)
- Household size
- 5Q-5D health-related quality of life
- High-risk status (offered flu vaccine)
- Current symptoms

- Vaccine confidence
- Public transport
- Distance travelled in past week

Participants will be asked to record the following information about their contacts:

- Time of contact
- Age
- Gender
- Geographical location (first part of postcode)
- Ethnicity
- Occupation
- Contact location (home/work/school/etc.)
    - Indoor/outdoor
- Duration of contact
- Physical/non-physical contact
- Is the contact a household member (yes/no)
- Frequency of contact (how many days out of the week do you typically interact with this person?)
- Attendance of large contact events
    - Option to record high numbers of contacts which would be difficult to record individually
- Contact with animals (pets, livestock (chickens, pigs, cattle, etc.))

Statistics and Data Analysis

Sample Size

We define an appropriate minimum sample size for this study as whether a difference in overall mean contacts can be detected compared to previous surveys conducted in GB / the UK - POLYMOD (Mossong et al., fieldwork 2005-6) and CoMix (Jarvis et al., final survey Nov-Dec 2022). There were substantial differences in the overall mean daily contacts reported in each of these two surveys (11.74 and 6.5, respectively) with contributing factors being the lasting impacts of the COVID-19 pandemic and rise of widespread digital communication and social media. Thus, we predict that overall contact rates will have decreased compared to POLYMOD, and increased compared to the final CoMix survey. As such, we specify a meaningful change as being at least a 10% decrease in contacts versus POLYMOD and at least a 10% increase versus the final CoMix survey. Another difference is that of the degree of overdispersion in daily contact rates; POLYMOD reported a theta of 2.78, though a secondary analysis of CoMix indicated much greater overdispersion (0.58; lower values indicating greater overdispersion).

With a sample size of at least 463 we will be able to detect a 10% decrease with 80% power compared to the POLYMOD contact survey conducted in 2005-6 (Table 1), assuming overdispersion parameter of 2.78; a sample size of at least 3517 is required to detect a difference compared to the final CoMix survey in 2022, assuming an overdispersion parameter of 0.58.

The final sample size will be subject to budget, though we will target 5000 in each seasonal round to reduce the risk of sparsity in the contact matrices. Quotas on age, sex, ethnicity, and geographic location will be used to ensure that a representative sample of the UK population is reached and exceeded for minority ethnic groups (target: to double the representation of non-white participants in the survey).

Table: Minimum sample sizes required to detect changes in overall contacts compared to previous surveys, given assumptions on magnitude of change and overdispersion.

Previous survey	Number of participants	Overall mean contacts	Relative change in number of reported contacts compared to previous survey	Dispersion parameter	Sample size required to achieve power
POLYMOD (2005-6)	1012	11.74	-10%	0.36	463
CoMix (final survey, Nov-Dec 2022)	2991	6.5	+10%	1.72	3517

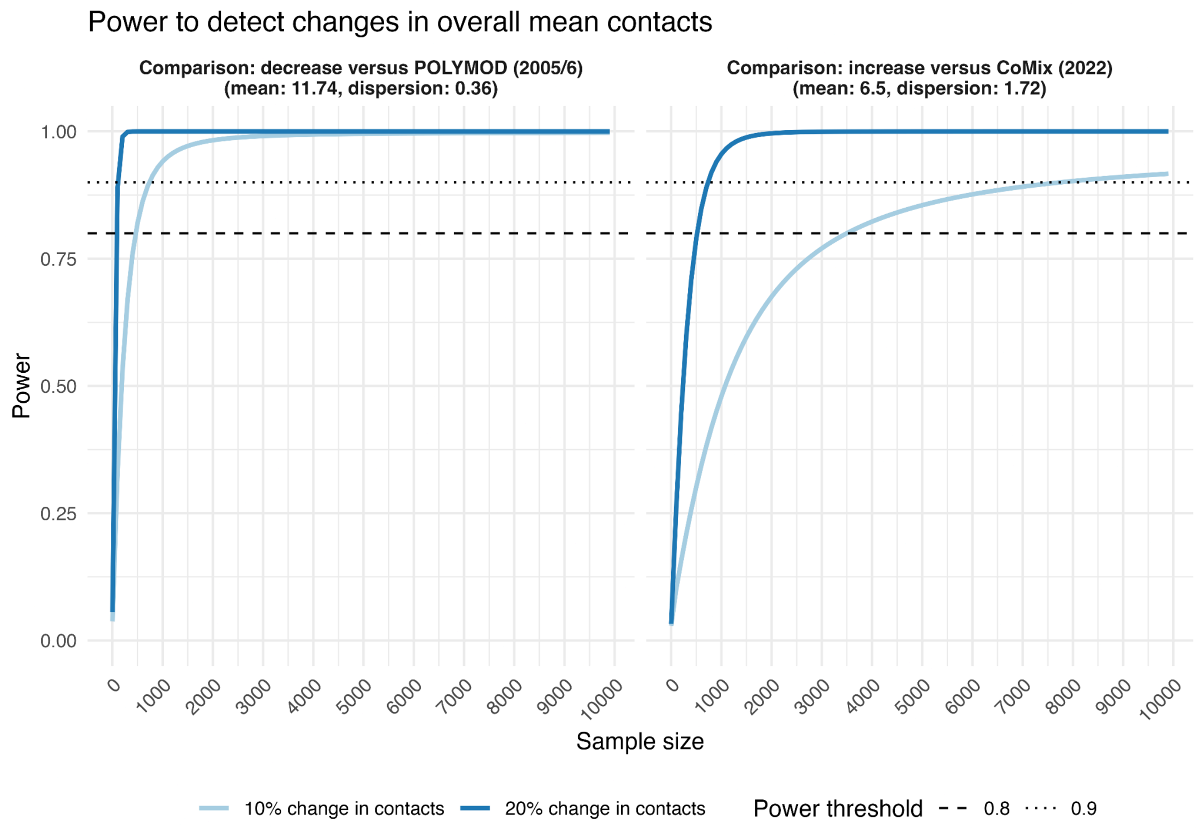

Figure: Required sample size to achieve power exceeding 0.8 and 0.9 for the comparisons of a change of -10% (or -20%) mean contacts versus POLYMOD and +10% (or +20%) mean contacts versus the final CoMix survey.

Statistical Analysis

Count models (negative binomial regression) will be employed to estimate the mean number of daily contacts and account for the likely substantial heterogeneity between individuals. These will be stratified by the different demographic characteristics of the participants to account and assess for any differences between groups. We will also develop social contact matrices considering assortativity of contact between different age groups, as well as investigate the additional dimensions of ethnicity, socioeconomic status, and other relevant factors. These estimates will inform ongoing and future response efforts to guide public policy decisions.

Regulatory Details

We will seek ethics approval from the LSHTM Research Ethics Committee, with additional approvals obtained where necessary. Informed consent will be obtained before participants complete the survey. Individuals will have the right to refuse to participate and withdraw from the survey if they wish, without needing to give a reason.

All data will be stored securely, ensuring confidentiality, following the Data Protection Act.

Dissemination of Findings

Dissemination of findings will occur via a research paper (initially as a preprint and eventually as a peer-reviewed publication. Anonymised data will be made publicly available to contribute to broader research efforts.

Changes to protocol

**Original protocol**	**Changes in final protocol**
**Study design**<br>“We will conduct several cross-sectional surveys in the United Kingdom in summer and in winter, covering holiday/non-holiday periods in each (term time/school holidays and pre-Christmas/ Christmas).”	**Study design**<br>Due to issues with the quality of the data collected during the late summer period in 2024, additional surveys were conducted over the autumn and winter period. As such, we were unable to make formal comparisons between these time periods; however, we report differences in contacts between school term-time and holidays by age group.
**Sample size**<br>The final sample size will be subject to budget, though we will target 5000 in each seasonal round to reduce the risk of sparsity in the contact matrices. Quotas on age, sex, ethnicity, and geographic location	**Sample size**<br>We conducted a cross-sectional social contact survey in the UK United Kingdom from 13th December 2024 to 10th February 2025. Participants were recruited from a UK-based internet panel, using quota

will be used to ensure that a representative sample of the UK population is reached and exceeded for minority ethnic groups (target: to double the representation of non-white participants in the survey).	sampling to ensure a close representation of the UK population in terms of gender, country, region (as defined by the Office for National Statistics (ONS), within England), household income, and education. We aimed to recruit at least 10,000 adult and 1,000 child participants, deliberately oversampling non-White participants and children in order to reduce data scarcity when conducting stratified analyses. A total sample size of 10,000 individuals was targeted over the duration of the study.<br>As recruitment rates for children and individuals aged 70+ were lower than expected, we conducted a second “boost” round of the survey from 22nd February to 10th March 2025 to increase representation in these age groups.
**Measurements**<br><br>Participants will be asked to record the following information about themselves:<br>● Age<br>● Gender<br>● Geographical location (first part of postcode)<br>● Ethnicity<br>● Household income<br>● Occupation<br>● Highest level of education<br>● Housing status (homeowner, private renter, etc.)<br>● Household size<br>● 5Q-5D health-related quality of life<br>● High-risk status (offered flu vaccine)<br>● Current symptoms<br>● Vaccine confidence<br>● Public transport<br>● Distance travelled in past week<br><br>Participants will be asked to record the following information about their contacts:<br>● Time of contact<br>● Age<br>● Gender<br>● Geographical location (first part of postcode)	**Measurements**<br><br>Participants were not asked to record contact with animals.

• Ethnicity<br>• Occupation<br>• Contact location (home/work/school/etc.)<br>○ Indoor/outdoor<br>• Duration of contact<br>• Physical/non-physical contact<br>• Is the contact a household member (yes/no)<br>• Frequency of contact (how many days out of the week do you typically interact with this person?)<br>• Attendance of large contact events<br>○ Option to record high numbers of contacts which would be difficult to record individually<br>• Contact with animals (pets, livestock (chickens, pigs, cattle, etc.))	
